# The biomechanical importance of the scaphoid-centrale fusion during simulated knuckle-walking and its implications for human locomotor evolution

**DOI:** 10.1038/s41598-020-60590-6

**Published:** 2020-02-26

**Authors:** Thomas A. Püschel, Jordi Marcé-Nogué, Andrew T. Chamberlain, Alaster Yoxall, William I. Sellers

**Affiliations:** 1 Primate Models for Behavioural Evolution Lab, Institute of Cognitive and Evolutionary Anthropology, School of Anthropology, University of Oxford, 64 Banbury Road, OX2 6PN Oxford, United Kingdom; 2 Center of Natural History (CeNak), Universität Hamburg, Martin-Luther-King-Platz 3, Hamburg, 20146 Germany; 3 Institut Català de Paleontologia Miquel Crusafont, Universitat Autònoma de Barcelona, Edifici ICTA-ICP, c/Columnes s/n, 08193 Cerdanyola del Vallès, Barcelona, Spain; 4 Department of Earth and Environmental Sciences, University of Manchester, M13 9PL Manchester, United Kingdom; 5 Art and Design Research Centre, Sheffield Hallam University, Sheffield, United Kingdom

**Keywords:** Biological anthropology, Biomechanics

## Abstract

Inferring the locomotor behaviour of the last common ancestor (LCA) of humans and African apes is still a divisive issue. An African great-ape-like ancestor using knuckle-walking is still the most parsimonious hypothesis for the LCA, despite diverse conflicting lines of evidence. Crucial to this hypothesis is the role of the centrale in the hominoid wrist, since the fusion of this bone with the scaphoid is among the clearest morphological synapomorphies of African apes and hominins. However, the exact functional significance of this fusion remains unclear. We address this question by carrying out finite element simulations of the hominoid wrist during knuckle-walking by virtually generating fused and unfused morphologies in a sample of hominoids. Finite element analysis was applied to test the hypothesis that a fused scaphoid-centrale better withstands the loads derived from knuckle-walking. The results show that fused morphologies display lower stress values, hence supporting a biomechanical explanation for the fusion as a functional adaptation for knuckle-walking. This functional interpretation for the fusion contrasts with the current inferred positional behaviour of the earliest hominins, thus suggesting that this morphology was probably retained from an LCA that exhibited knuckle-walking as part of its locomotor repertoire and that was probably later exapted for other functions.

## Introduction

As noted by Darwin^1^, bipedalism with an upright posture is probably the main defining trait of the earliest hominins. This locomotor behaviour greatly contrasts with those observed in other African apes, which mainly exhibit diversified quadrupedal locomotor behaviours^2^. Using the extant great apes as analogues, several different locomotor modes have been advanced to characterise the ancestral condition prior to the adoption of the strict bipedal locomotion seen in the human lineage^3,4^. Among the ancestral proposed locomotor modes, knuckle-walking, a characteristic terrestrial quadrupedal locomotion exhibit by African apes, has played a central role. This is because it represents the most parsimonious of the alternative hypotheses since both *Pan* and *Gorilla* exhibit this locomotor behaviour. The knuckle-walking hypothesis states that the last common ancestor (LCA) of gorillas, chimpanzees and humans possessed this locomotor mode, whereas vertical climbing, a common behaviour observed in all of the extant apes, would be considered ancestral to knuckle-walking^5,6^. Nevertheless, both phyletic and functional analyses of the fossils of *Ardipithecus ramidus* suggest that hominin bipedality might have evolved from a locomotor mode that does not have a modern analogue among the great apes (i.e., careful climbing, clambering, and bridging)^7–9^. In addition, some authors point out that knuckle-walking could have evolved independently in the African great apes, which would imply an homoplasic evolution of this locomotor behaviour in *Gorilla* and *Pan*^10,11^. Other studies have argued the opposite by pointing out that the observed differences in knuckle-walking between these two genera can be explained by differences in positional behaviour, kinematics, and the biomechanics of weight-bearing^12,13^, without implying two independent origins of knuckle-walking. Additionally, a recent re-assessment of the foot morphology of *Ardipithecus ramidus* suggests that the LCA probably exhibited an African ape-like locomotor repertoire involving terrestrial quadrupedalism and climbing^14^. Due to the importance of this matter, several studies have focused on the analysis of the hominoid wrist since it represents the key anatomical location that could help to elucidate whether the LCA was a knuckle-walker or not (for a summary see^15^).

Among mammals, the wrist is arguably one of the most complex joint systems, comprising around 15–17 bones, which are connected by at least 20 articulations, and bound together by several tendons and ligaments^15^. Despite being composed of numerous elements, the carpal bones work together to transfer loads between the hand and forearm, thus enabling the mobility of the hand in multiple planes^16^. Interestingly, among the scarce osteological synapomorphies of humans and African apes, the fusion of the os centrale to the scaphoid has been long recognised^17,18^. The scaphoid-centrale fusion in the African hominoid carpus has been interpreted as a functional adaptation to the stresses exerted on this joint during quadrupedal locomotion, particularly accentuated in knuckle-walking. The functional hypothesis has led to the suggestion that the fusion is evidence for a knuckle-walking common ancestor of the hominine clade^6^. In modern humans, the persistence of this feature has been interpreted as either a phylogenetic vestige^19^, or an exaptation to withstand the shear stress during power-grip positions^20^. Among other anthropoids, *Pongo* sometimes exhibits this feature, but only African apes and fossil and extant humans show this trait in almost all individuals^18^.

Currently, the predominant functional hypothesis explaining the evolution of the fusion of the os centrale and the scaphoid considers that it decreases mobility and improves the ability to transmit forces between the manual rays and radius during knuckle-walking^16^, hence its importance when inferring the locomotor mode of the LCA^17,20,21^. This is especially important in African apes, because they do not have a weight-bearing articulation between the carpals and the ulna, so they have to use a hand posture in which ground reaction forces travel along metacarpals, carpals, and the radius^21^. This hypothesis has found some support in the convergent evolution of carpal fusion in other knuckle-walking species such as giant anteaters^22^, as well as chalicotheres^5,17^. However, it is also known that some lemurs, including suspensory sub-fossils such as *Palaeopropithecus*, also show the fusion of the os centrale^23,24^. This observation led some researchers to discard the knuckle-walking hypothesis for the os centrale fusion in the African hominoid clade^25^. Nonetheless, others consider that lemur wrist structure and function are sufficiently dissimilar to have evolved for different reasons^5^. For this reason, some argue that neither the functional nor the phylogenetic significance of scaphoid-centrale fusion are completely clear^18^, and that further analyses are required to establish whether this carpal fusion represents a significant functional adaptation. Fortunately, the functional virtual morphology toolkit allows us to generate fused and unfused morphologies to test this using a representative hominoid sample. Consequently, the present work uses finite element analysis (FEA) to assess whether the scaphoid-centrale fusion improves the transmission of forces between the manual rays and radius. If the fusion of the centrale to the scaphoid acts biomechanically to limit stresses as would be expected as an adaptation to knuckle-walking, then higher stresses should be expected in those specimens with an unfused centrale (either naturally or virtually unfused morphologies). However, it is important to keep in mind that functional homology does not necessarily imply structural homology (i.e., caution is required when assessing the results since having the same function does not necessarily mean that the structures, as opposed to the functions, are homologous). In this sense, if a functional explanation for the fusion that is incompatible with the current inferred positional behaviour of the earliest hominins is found (i.e., fused morphologies better withstand stresses derived from knuckle-walking), then this would suggest that the fused morphology was retained from a LCA that exhibited knuckle-walking as part of its locomotor repertoire, and that then was later exapted for other functions.

## Methods

### Model properties

**Figure 1:**
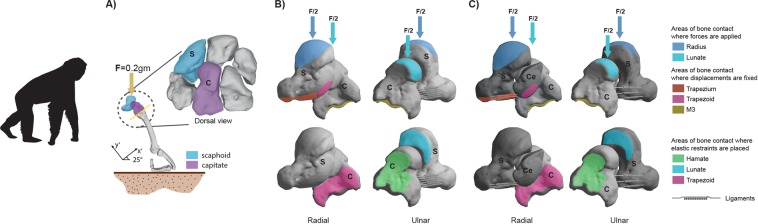
Biomechanical problem under analysis displayed using the bones from the left limb of a *Pan troglodytes* specimen (S: scaphoid; C: capitate; Ce: centrale. (**A**) Depicts the position of the bones under analysis during a standing scenario, (**B**) shows a fused model and (**C**) displays a non-fused model (i.e., the scaphoid and centrale are simulated as separated bones). Please note that view of the carpal bones was defined according to the human anatomical standard position.

CT-scans of the wrists of adult individuals belonging to different hominoid species were obtained from Digital Morphology Museum, KUPRI (http://dmm.pri.kyoto-u.ac.jp/dmm/WebGallery/index.html), whilst the human individual was downloaded from Morphosource (https://www.morphosource.org/) (further details about the sample can be found in the electronic Supplementary Material S1). The included species were *Hylobates lar*, *Pongo abelii*, *Gorilla gorilla*, *Pan troglodytes*, and *Homo sapiens*. Virtually reconstructed surfaces of each specimen were created with Seg3D version 2.1.5 (CIBC, USA) where each specimen was segmented by applying a combination of case-specific thresholding values and manual painting techniques. We virtually generated fused and unfused morphologies of our entire sample depending on the species to compare biomechanical performance (further details about this process are provided in S2). The unfused morphologies comprised three bony elements (i.e., capitate, scaphoid and centrale) connected by the scaphocentralecapitate ligament, whereas the fused morphology involved the scaphoid (or fused scaphoid-centrale) and capitate connected by the scaphocapitate ligament. The segmented models were then converted to CAD models and oriented with respect to the same plane to facilitate the comparison between them as shown in Fig. 1A.

A structural static analysis to evaluate the biomechanical behaviour of the scaphoid, capitate and centrale during the stance phase of knuckle-walking was performed using ANSYS® (Ansys Inc., version 17.1, Canonsburg, PA). Elastic, linear, and homogeneous material properties were assumed for both capitate and scaphoid in the fused models and, capitate, centrale and scaphoid for the non-fused ones. The following mechanical properties were assumed for cortical bone (Young Modulus E = 10 GPa and Poisson’s ratio v = 0.22), cancellous bone (Young Modulus E = 0.5 GPa and Poisson’s ratio v = 0.3) and the cartilage that surrounds the bones (Young Modulus E = 7 MPa and Poisson’s ratio v = 0.3)^26^. See Fig. S3 for the different parts of the model.

Shell elements were used to mesh the cartilage and cortical bone, whereas solid three-dimensional tetrahedral elements were used to mesh the cancellous bone. The shell elements defining the cortical bone and the solid elements defining the cancellous bone were connected using a multipoint constraint approach where the degrees of freedom of both types of elements are totally coupled. As shell elements, the thickness of the cartilage and the cortical bone were assumed to be constant (Table S1). The bone values were directly measured from the CT data, whereas cartilage thickness was assumed to be 0.55 mm since this data is not easily available. The articular surfaces of real joints tend to be irregular and cartilage thickness varies as a function of the specific location. Such variations cannot be effectively simulated with simplistic geometric representations of articular joints; therefore, we chose this conservative value to accommodate the possible variations in thickness for all the analysed species, based on data published for humans^27,28^. The mesh of the analysed models ranged approximately between 1.5 and 3 million tetrahedral elements and 30,000–50,000 shell elements depending on the model (See S4 for further details).

### Ligaments

Linear spring elements were used to model the ligaments with their locations being estimated from previous anatomical studies^16^. The origins and the insertion points were distributed over an area by using multiple springs in parallel with each other. Fig. 1 shows the ligament configuration for the model.

The rigidity of the ligament was assumed as 40 N/mm based in the values from^29^ for the palmar scaphocapitate carpal ligaments. The values of each spring in the model were set according to the parallel and serial distribution of the springs in the fused and non-fused cases (Table S5).

### Boundary and loading conditions

Hominoid forelimbs support about 40% of the body weight during terrestrial quadrupedalism^30^. Hence, the total applied load was calculated as 20% of the body mass multiplied by gravitational acceleration (*g*: 9.81 ms^−2^) (Table 1). Body mass was obtained from^31^, excepting for *H. lar* for which the individual data was available. The load was applied on the radial articular surface of the scaphoid and on the lunate articular surface of the capitate (Fig. 1B,C).

**Table 1:** Body mass and applied loads for the individuals under analysis.

Model	Body mass [Kg]	Total load [N]
*H. sapiens*	72.1	141.41
*G. gorilla*	170.4	334.21
*P. troglodytes*	59.7	117.09
*P. abelii*	77.9	152.79
*H. lar*	9.5	18.63

Fixed displacements were placed on the lower portion of the model in the areas of the scaphoid (centrale for the non-fused) and capitate where these bones meet the third metacarpal, the trapezoid and the trapezium (Fig. 1B,C). Lateral movements were partially restrained on the contact areas of the model with the lunate, hamate and trapezoid (Fig. 1B,C). This partial restriction was based on the fact that the contact with these bones is mediated by articular cartilage and, therefore, the model elasticity allows the scaphoid and the capitate to move laterally. To create the partial restraint, we applied a lateral elastic boundary condition based on a cartilage carpal stiffness of 20.5 N/mm as defined in^32^. The contact between the outer surfaces of the cartilages of the model (scaphoid and capitate in the fused models and scaphoid, centrale and capitate in the non-fused models) was defined as a non-separation contact which enables sliding on the plane of the contact and that does not allow a normal separation between bodies in the perpendicular direction.

### von Mises stress

We obtained the von Mises stress distribution in the carpal elements under the chosen loading conditions. The von Mises stress distribution was evaluated using their average values and presented using box-plots to display their stress distribution. This required the use of a quasi-ideal mesh (QIM), which is a non-uniform mesh (i.e. different elements have dissimilar sizes, although nearly identical in a QIM), which in turn needed the use of recently proposed statistics that take into account this non-uniformity (i.e., the mesh weighted arithmetic mean [MWAM], and the mesh-weighted median [MWM])^33^. The obtained stress results for the scaphoid and capitate from the fused and non-fused models followed a normal distribution based on a Shapiro-Wilks test (Scaphoid: W = 0.96811, p-value = 0.8728, n = 10; Capitate: W = 0.89857, p-value = 0.2113, n=10). Therefore, they were compared using Welch’s two sample t-tests in R version 3.5.1^34^.

Additionally, simulations considering a force applied at 5° were also carried out to test if changing the direction of the applied force could have had an important impact in our results. The obtained results from this additional simulation set do not differ significantly from our main set of simulations (Scaphoid: t = −0.063888, df = 17.996, p-value = 0.9498; Capitate: t = −0.02848, df = 18, p-value = 0.9776). Further details about this additional simulation set can be found in S6, whereas the numerical results are provided in S7.

## Results

**Figure 2:**
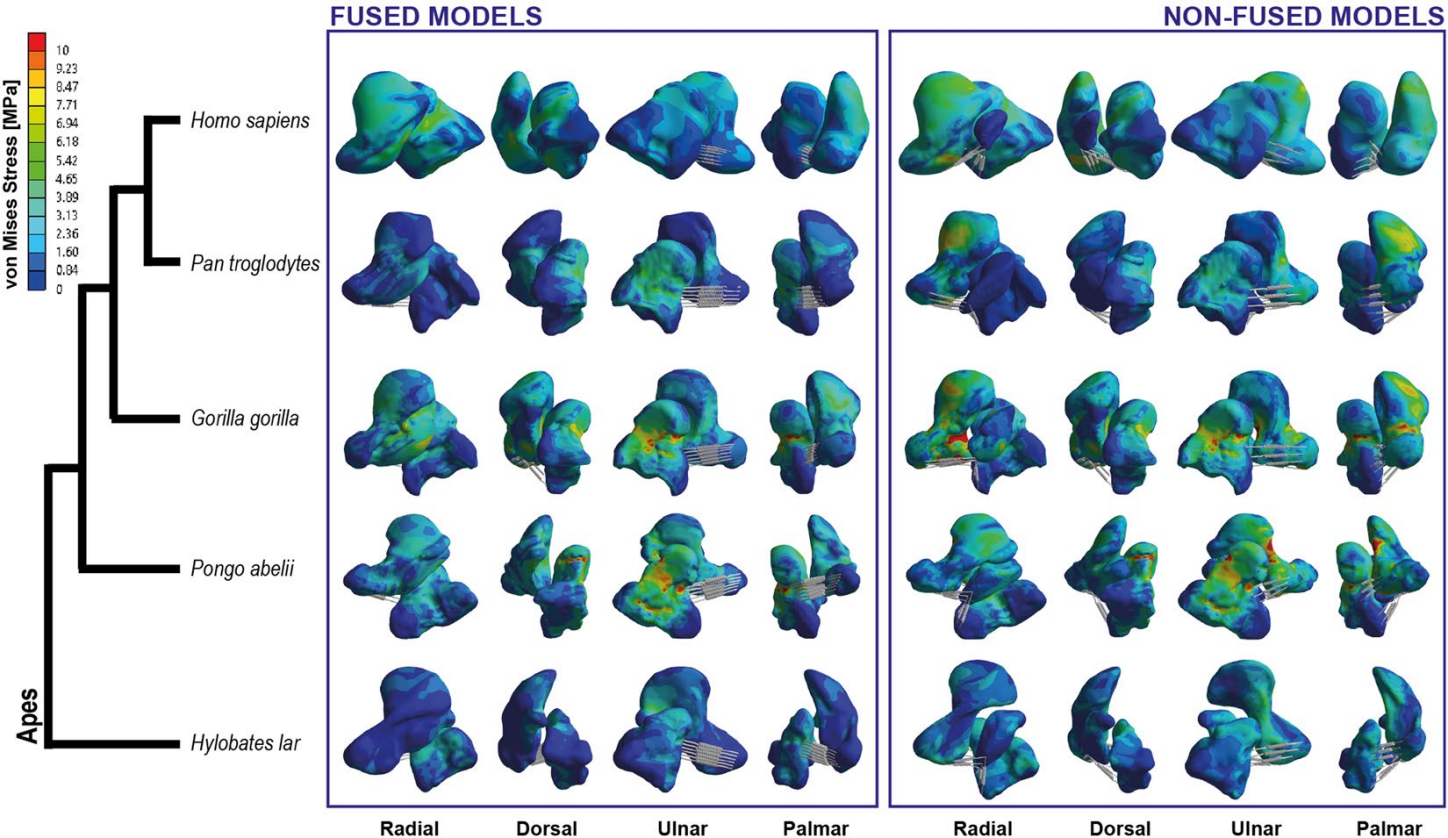
von Mises stress distribution of the analysed sample for both the fused and non-fused models. For simplicity, views were defined according to the human anatomical standard position.

**Figure 3:**
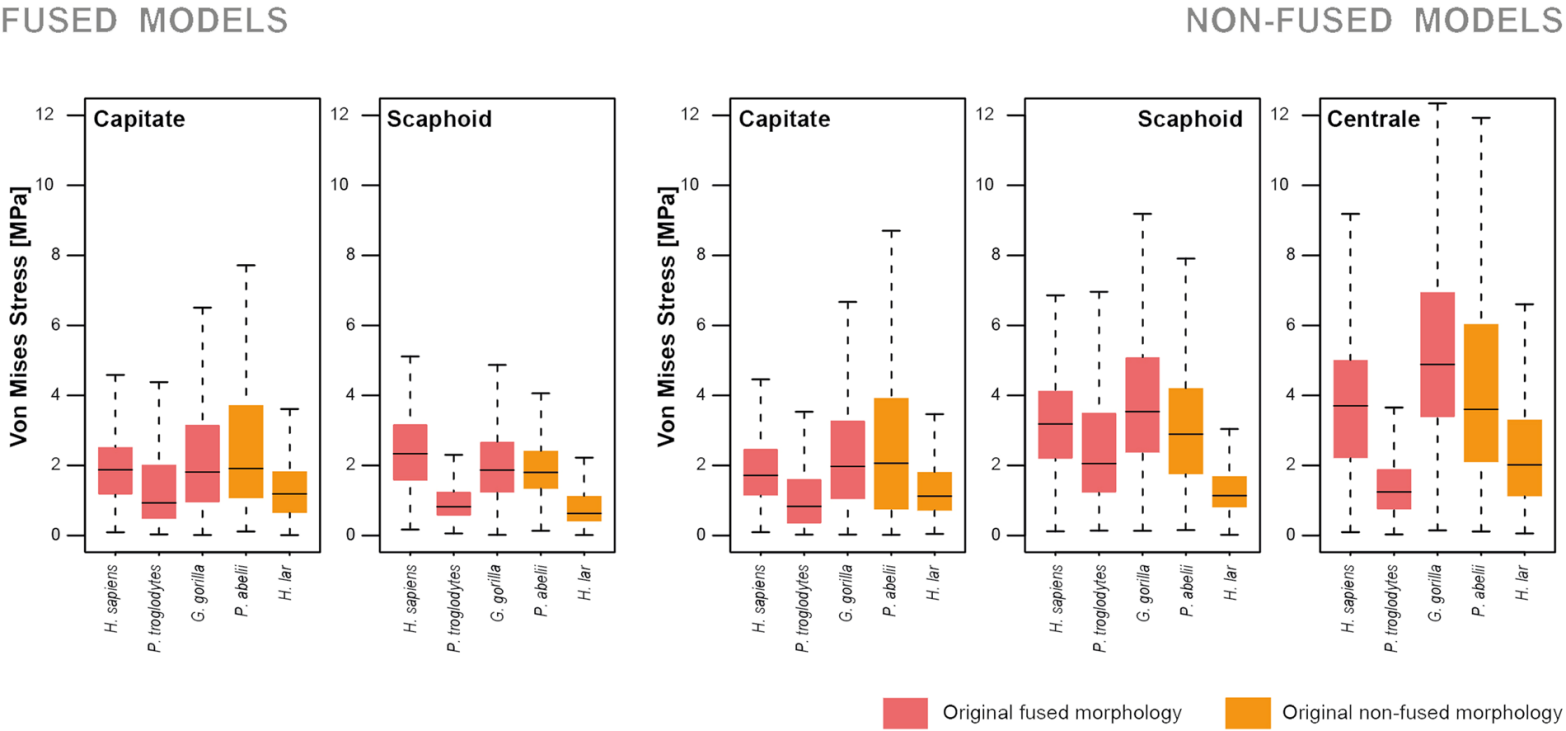
Boxplots of the stress distribution for both the fused and non-fused morphologies for all the species under analysis. The summary statistics used to create the boxplot are the median of the data (line), the lower (25%) and upper quartiles (75%) (box limits) and the minimum and maximum values (whiskers).

The visual representation of the von Mises stress distributions is provided in Fig. 2, whereas Fig. 3 shows the stress distribution of the QIM in boxplots. These figures show that fused models exhibit noticeable lower stress values as compared to the non-fused models. This is confirmed for the scaphoid models when comparing their stress values (i.e., MWAM). The fused scaphoid models (n=5) showed significantly lower stress values as compared to the non-fused ones (n=5) t = −2.4534, df = 7.5262, p-value = 0.04155). However, there were no significant differences for the capitate stress values (t = 0.13753, df = 7.8102, p-value = 0.8941). The capitate showed the lowest stress levels, whilst the centrale experienced the highest ones. All the stress values obtained as result from our simulations are available in the Supplementary Material S7.

## Discussion

The results presented here support the biomechanical aspect that is required to interpret the fusion of scaphoid and the centrale as a functional adaptation for knuckle-walking. The biomechanical results show that fused morphologies better withstand the simulated loading scenario as compared to the non-fused models. All fused morphologies (i.e., both natural as well as those virtually generated) exhibited lower stress values when compared to the non-fused ones. Thus, these results show from a mechanical perspective that the fused morphology is better at withstanding the forces derived from knuckle-walking. When we virtually separated the centrale from the scaphoid, it seems that we removed a morphological portion of the bone that was necessary to better distribute the stresses derived from the applied external forces. Another possibility is that the lack of a cartilage layer could improve the force coupling, preventing the stress concentration that might be caused by the thin layer of dissimilar material. In any case, fused scaphoid-centrales show less stress as compared to unfused morphologies.

It is important to keep in mind some of the limitations of the present study that could influence our results. For instance, we used the same material properties of cancellous and cortical bone, cartilage and ligaments for all the studied species, and we also assumed homogeneity. These values were obtained from humans and applied to all the models because there is no available information of these values for the other analysed apes. Moreover, the use of homogeneous material properties for the bone is also widely accepted in the community, especially when modelling carpal bones since there is no accurate published information available for all the analysed species^35^. We simplified the models by defining constant thickness for the cartilage and the bone. This is probably not the best ideal option, but the lack of available information did not allow us to generate more sophisticated models. We modelled the cartilage as a layer covering the whole body of the bone (i.e., as an additional layer). This also represents an additional simplification because cartilage does not really cover the totality of the bone. Nonetheless, this simplification does not influence the stress results observed in the bone. In fact, since we are simply comparing the fused and unfused models from the same species with respect to each other, we would expect a minimal impact assuming all these limitations and simplification from a comparative point view.

It has been argued that knuckle walking was adopted by the African apes as a way to ameliorate the consequences of repetitive impact loadings on both soft and hard tissues of the forelimb via isometric and/or eccentric contraction of the antebrachial muscles during terrestrial locomotion^36^. Our obtained results are consistent with this interpretation, since they show that the scaphoid-centrale fusion plays a role in reducing the experienced loads during knuckle-walking, which suggests that high loads due to quadrupedal locomotion were indeed a problem and a skeletal trait that contributes to better withstand the mechanical loads from knuckle-walking would therefore be adaptive. This result also provides some functional support for the hypothesis that considers the LCA as a knuckle-walker, although, of course, it does not prove it. As mentioned before, it is important to keep in mind that functional homology does not necessarily imply structural homology (i.e., caution is required when assessing the observed results since having the same function does not necessarily mean that the structures, as opposed to the functions, are homologous). However, the fact that the functional explanation for the scaphoid-centrale fusion contrasts with the inferred positional behaviour of the earliest hominins, thus suggests that this morphology was probably retained from an LCA that exhibited knuckle-walking as part of its locomotor repertoire and that was later exapted for other functions.

Consequently, the biomechanical results obtained here are consistent with more classic interpretations^20,37^ that consider the scaphoid-centrale fusion as a functional adaptation to better resist weight-bearing stresses and increase the stability of the hand in terrestrial quadrupedalism. This interpretation is in agreement with other traits shared between African apes and humans, such as robust metacarpals and phalanges and long middle phalanges relative to proximal phalanges, which might also represent knuckle-walking adaptations. In addition, other studies that focused on ligaments and kinematics have also shown some support to the hypothesis that the scaphoid-centrale fusion contributes to a more rigid mid-carpus in the African apes in a way that may stabilises the wrist during knuckle-walking^16^. This work is also concordant with previous morphological studies, which have suggested that several radial traits in the African apes are involved in stabilizing the wrist in extension during knuckle-walking^6^. The obtained results in our study are also consistent with a recently published study that analysed the oldest-known fossil foot (4.4 myr) attributed to *Ardipithecus ramidus*^14^. This study of the foot used evolutionary models to assess the relationship between tarsal proportions and the locomotor behaviour of both apes and monkeys and suggests that hominins evolved from an ancestor that had a foot similar to the African apes, thus suggesting an LCA with an African ape-like foot adapted to terrestrial plantigrade quadrupedalism and some climbing^14^.

Nevertheless, it is important to consider that this conclusion would also require the explanation of diverse conflicting lines of evidence and opposing interpretations of the available data. For instance, it has been strongly argued that some aspects of the hominoid carpus morphology^10,11^, great ape locomotor biomechanics^2^, as well as the current functional interpretation of *Ardipithecus ramidus* anatomy^8,9^ do not favour a knuckle-walking LCA, but rather the homoplasic evolution of this behaviour in chimpanzees and gorillas (but see^12,14^ for opposite opinions). In addition, another possible interpretation for the retention of scaphoid-centrale fusion in the bipedal hominin lineage might be attributable to exaptation, rather than phylogenetic legacy, as stresses in the wrist from activities such as tool making, or tool use might have been substantial. For example, it seems that in Oldowan knapping the highest forces are reported to be transmitted through the digits 2 and 3, hence likely onto the capitate. Whether stone tool behaviours could have led to the retention of the scaphoid-centrale fusion requires further investigation.

Consequently, the functionally adaptive nature of the scaphoid-centrale fusion has to be shown at different levels to be fully corroborated, since the present paper exclusively shows its functional role^18,38^. Fossil evidence linking the origins of knuckle-walking with the appearance of scaphoid-centrale fusion would be able to clarify the adaptive significance of this trait. In addition, further biomechanical models and experimental data on this issue can also contribute in elucidating the role of the scaphoid-centrale fusion. For example, by analysing unrelated taxa (e.g., ground sloths, giant pandas, chalicotheres, sub-fossil lemurs, giant anteaters, etc.) and exploring the functional interpretations of fused and non-fused wrist bones within different phylogenetic and locomotor contexts, we should be able to further clarify the functional role of this trait. Finally, it is important to keep in mind that extant knuckle-walkers show diverse positional behaviors, and that knuckle-walking does not preclude climbing or exclude the possible importance of arboreality in the evolution of bipedalism in our lineage.

## Supplementary information

Supplementary information.

## Data Availability

Further details about the article can be found as part of electronic supplementary material. Original CT scans of all specimens are available from http://dmm.pri.kyoto-u.ac.jp/ and https://www.morphosource.org/. Accession numbers are provided in Table S1.
