# Supplementary material for: The biomechanical importance of the scaphoid-centrale fusion during simulated knuckle-walking and its implications for human locomotor evolution
Source: Sci Rep. 2020 Feb 26;10:3526. doi: 10.1038/s41598-020-60590-6 (PMC7044280; doi:10.1038/s41598-020-60590-6)
Supplement: Supplementary file 1 — Supplementary information. [file 41598_2020_60590_MOESM1_ESM.pdf]

Supplementary information

The biomechanical importance of the scaphoid-centrale fusion during simulated knuckle-walking and its implications for human locomotor evolution

Thomas A. Püschel[1]*, Jordi Marcé-Nogué[2,3], Andrew T. Chamberlain[4], Alaster Yoxall[5], William I. Sellers[4]

1 Primate Models for Behavioural Evolution Lab, Institute of Cognitive and Evolutionary Anthropology, School of Anthropology, University of Oxford, 64 Banbury Road, OX2 6PN, Oxford, United Kingdom.
2 Center of Natural History (CeNak), Universität Hamburg, Martin-Luther-King-Platz 3, Hamburg, 20146, Germany.
3 Institut Català de Paleontologia Miquel Crusafont, Universitat Autònoma de Barcelona, Edifici ICTA-ICP, c/ Columnes s/n, 08193 Cerdanyola del Vallès, Barcelona, Spain.
4 School of Earth and Environmental Sciences, University of Manchester, M13 9PL, United Kingdom.
5 Art and Design Research Centre, Sheffield Hallam University, Sheffield, United Kingdom.

*Corresponding author: Thomas A. Püschel
E-mail: thomas.puschelrouliez@anthro.ox.ac.uk

S1. Further sample details

Species	Common name	Online Database	Accession number	ID	Sex	Institution	Ontogenetic stage	Body mass (kg)
Gorilla gorilla	Western Gorilla	KUPRI	PRICT No. 1353	Yamato (GAIN 0064)	Male	Kobe Oji Zoo, Japan	Adult (38 years)	170.4*
Pongo abelii	Sumatran Orangutan/Mawas	KUPRI	PRICT No. 516	Baran (GAIN 8)	Male	Primate Research Institute, Kyoto University, Japan	Adult (49 years)	77.9*
Pan troglodytes	Common Chimpanzee	KUPRI	PRICT No. 319	Rick (GAIN 323)	Male	Primate Research Institute, Kyoto University, Japan	Adult (22 years)	59.7*
Hylobates lar	Lar Gibbon/White-handed Gibbon	KUPRI	PRICT No. 465	Kuro	Male	Primate Research Institute, Kyoto University, Japan	Adult (33 years)	9.5
Homo sapiens	Modern human	Morphosource	P419	-	Male	Jan Palfijn Anatomy Lab, KU Leuven Campus Kulak, Kortrijk, Belgium	Adult (60 years)	72.1*

*body mass from Smith & Jungers (1997)

S2. Protocol followed to virtually 'unfuse' the centrale from the scaphoid.

Virtually reconstructed surfaces of each specimen were created with Seg3D version 2.1.5 (CIBC, USA) where each specimen was segmented by applying a combination of case-specific thresholding values and manual painting techniques. Surfaces were then generated and exported as .STL files into Geomagic Studio v. 12 (Geomagic, USA). Using this software, possible errors in the polygon mesh were detected and corrected to remove protruding vertices and localized holes. All the models were globally remeshed to simplify their element geometry. The remeshing process was applied to generate a more homogenous mesh in terms of the shape of the triangles, their distribution on the surface, and their connectivity.

Afterwards, using the same software (Geomagic Studio v. 12) a series of procedures were applied to separate the centrale from the scaphoid. In order to virtually 'unfuse' these two carpal bones, both the unfused models from *Pongo* and *Hylobates*, as well as the figures available in [1] were used as reference material. Some of the figures show the scaphoid and centrale of some the analysed genera at different ontogenetic stages (i.e., at different moments of the fusion process). In adult African apes and humans, the centrale is fused to the distomedial aspect of the scaphoid. Consequently, we traced the fusion line between centrale and the scaphoid using the 'custom region' selection tool, which allowed us to demarcate the centrale. Subsequently, using the 'expand' and/or 'shrink' selection tools, the selected area was expanded or reduced if necessary, to better define the centrale morphology. Once the selected area representing the centrales was determined, the model was duplicated to generate an additional copy. The area selected in one of the copies was reversed to select the scaphoid. Then the selected polygons were removed, thus resulting in two separated models (i.e., centrale and scaphoid) each one with a hole in the area where they used to be fused to each other. The 'fill single hole' tool was used to generate five artificial 'bridges' between the opposite edges of the holes of each one of the models using the 'bridge' and 'tangent' parameters. The 'bridge' option was preferred because it specifies that a bridge is built across a hole, thus dividing it into separately fillable holes. This enables to divide the holes of each one of the models into smaller ones that could be filled more correctly. The 'tangent' parameter ensured that the newly generated mesh filling the hole matched the curvature of the surrounding mesh, but with more tapering than the 'curvature' option. As result of this procedure, the holes located in the area where the articular surfaces between the scaphoid and centrale would have been, were divided into four smaller holes that were subsequently filled using the 'fill single' option. The same 'tangent' parameter was used, although this time we specified that the whole openings were filled. As an additional step we used the 'sandpaper' tool to smooth the margins of the obtained articular surface (parameters: 'relax operation' and 'medium strength'). A similar (but inverse) procedure was performed when fusing the models of *Hylobates* and *Pongo*. All the obtained models were then exported as .OBJ files into Autodesk 3ds Max 2012 (AutoDesk, USA), where they were converted into .SAT files prior to being imported into Ansys (Ansys Inc., version 17.1, Canonsburg, PA).

References

1. Kivell, T. L. & Begun, D. R. Frequency and timing of scaphoid-centrale fusion in hominoids. *J. Hum. Evol.* **52**, 321–340 (2007).

S3. Further details about the model

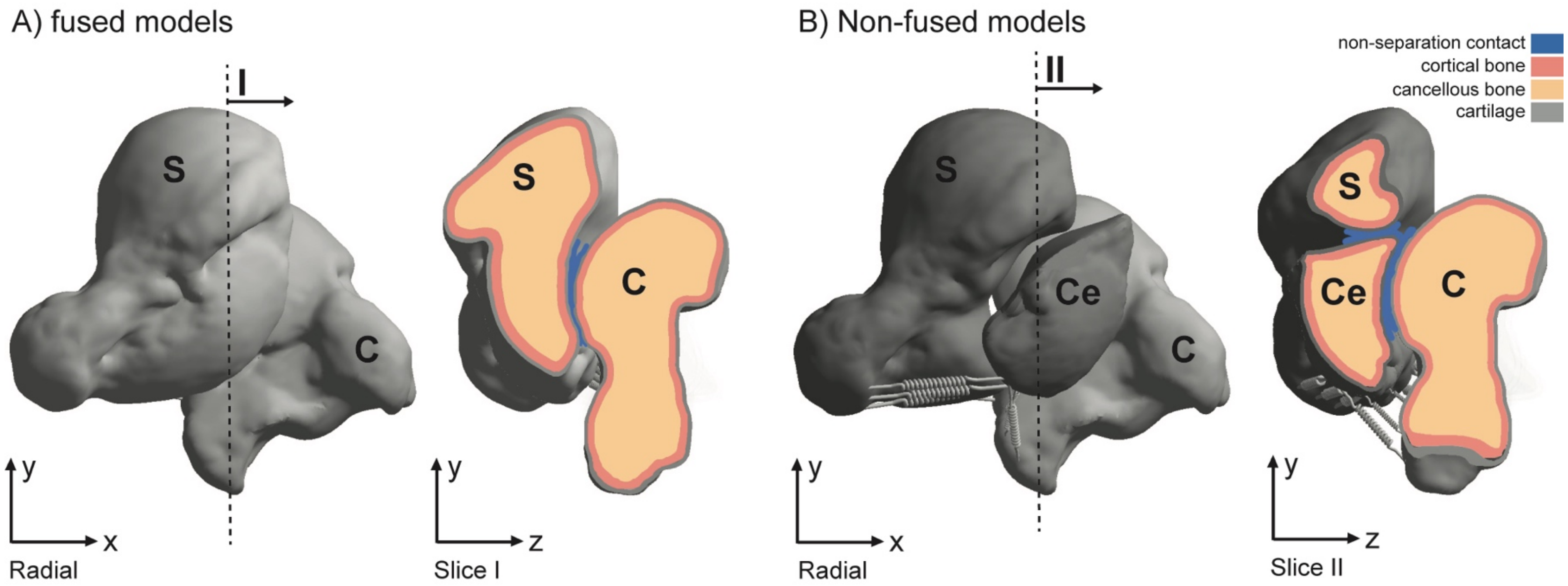

S4. Number of mesh elements

	Fused models			Non fused models		
**Species**	**Solid**	**Shell**	**Total**	**Solid**	**Shell**	**Total**
H. sapiens	2214575	32550	2247125	1629466	27801	1657267
G. gorilla	1979942	33659	2013601	2003834	37122	2040956
P. troglodytes	2316909	37922	2354831	2947256	49904	2997160
P. abelii	2816719	46339	2863058	2810745	50642	2861387
H. lar	1866544	34515	1901059	1847660	38079	1885739

S5. Configuration of the parallel-serial systems of springs for the ligaments.

Model	Fused models		Non-fused models			
	K_i [N/mm] 6 springs Scaphoid-capitate	K [N/mm] Total value	K_i [N/mm] 3 springs Scaphoid-capitate	K_i [N/mm] 3 springs Scaphoid-centrale	K_i [N/mm] 3 springs centrale-capitate	K [N/mm] Total value
H. sapiens	6.66	40	6.66	3.33	3.33	40
G. gorilla	6.66	40	6.66	3.33	3.33	40
P. troglodytes	6.66	40	6.66	3.33	3.33	40
P. abelii	6.66	40	6.66	3.33	3.33	40
H. lar	6.66	40	6.66	3.33	3.33	40

S6. Additional set of simulations carried out to test the possible influence of a slight change in the direction of the applied loading (5°).

Figure S6. 1 shows the applied loading scenario. The only difference when compared to the simulations described in the main text is that the load was applied at 5°.:

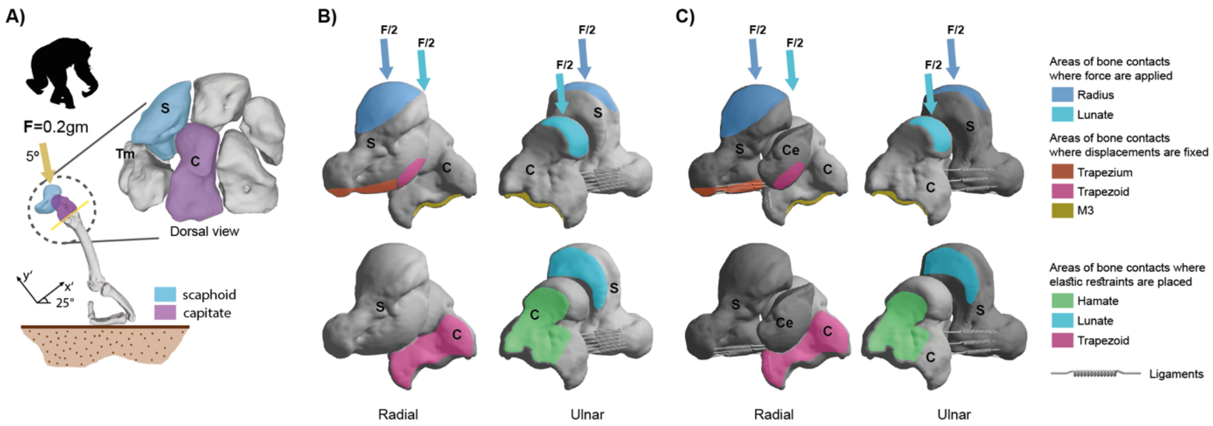

Figure S6.1 Biomechanical problem under analysis displayed using the bones from the left limb of a *Pan troglodytes* specimen (S: scaphoid; C: capitate; Ce: centrale. a) depicts the position of the bones under analysis during a standing scenario, b) shows a fused model and c) displays a non-fused model (i.e., the scaphoid and centrale are simulated as separated bones). Please note that view of the carpal bones was defined according to the human anatomical standard position.

Figure S6.2 shows the obtained results:

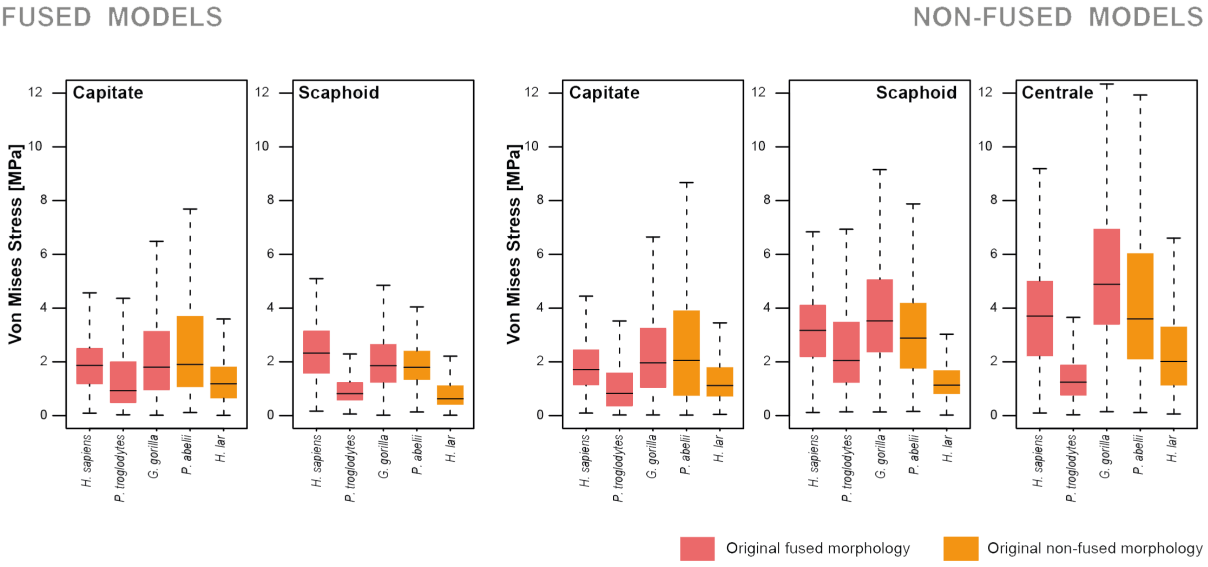

As it possible to notice, the observed pattern does not significantly differ from the one observed in Figure 3. When comparing the results between the two sets of simulations using a Kruskal-Wallis test, we obtained that there are no significant differences: vertical load vs. load at 5° (Scaphoid: t = -0.063888, df = 17.996, p-value = 0.9498; Capitate: t = -0.02848, df = 18, p-value = 0.9776).

Figure S6.3 shows the von mises stress distribution of the analysed sample for both the fused and non-fused models when the load was applied at 5°. The pattern is almost identical to the one observed in Figure 2. For simplicity, views were defined according to the human anatomical standard position.

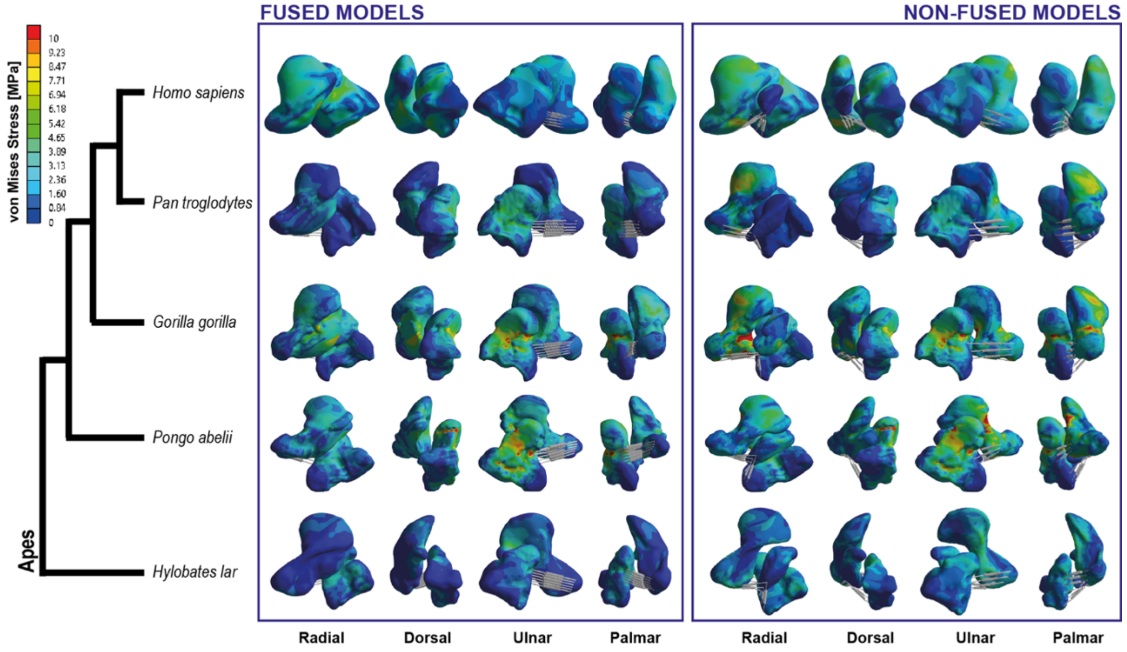

S7. Stress values obtained from the simulations

Species	Class	Simulation set	Number of elements	MWAM	MWM	M25	M50	M75	M95	PEof AM	PEofM
**Capitate**											
Gorilla gorilla	Fused	vertical	15959	2.20601937	1.74869258	0.9375225	1.8119	3.16865	5.37968	0.36295486	3.61455306
Pan troglodytes	Fused	vertical	18732	1.35364076	0.90097804	0.46492	0.929505	2.03215	3.67886	0.20955505	3.16622098
Homo sapiens	Fused	vertical	18267	1.90439018	1.78637395	1.1657	1.8757	2.534575	3.580745	0.14609155	5.00041172
Hylobates lar	Fused	vertical	14186	1.27889688	1.14039694	0.6427	1.1873	1.8328	2.61884	0.35635856	4.11287166
Pongo abelii	Fused	vertical	25065	2.50842916	1.8159891	1.06085	1.911	3.724825	6.003925	0.19160091	5.2319094
Gorilla gorilla	Non-fused	vertical	16069	2.31235664	1.86094602	1.027725	1.972	3.28675	5.4665	0.17207555	5.96760907
Pan troglodytes	Non-fused	vertical	21714	1.06613044	0.78258477	0.33621	0.830855	1.6174	2.87724	0.13576579	6.16805171
Homo sapiens	Non-fused	vertical	14854	1.82744711	1.6537399	1.1279	1.71765	2.4779	3.48106	0.46411856	3.86457989
Hylobates lar	Non-fused	vertical	14118	1.29047427	1.08955858	0.70976	1.12285	1.812	2.80246	0.82167485	3.05549587
Pongo abelii	Non-fused	vertical	25082	2.50272721	1.98599946	0.73595	2.06355	3.932	5.94244	0.18946592	3.90486218
Gorilla gorilla	Fused	five-degrees	15959	2.19783163	1.74162755	0.9340475	1.8051	3.15615	5.35811	0.3627358	3.64443302
Pan troglodytes	Fused	five-degrees	18732	1.34832319	0.89743902	0.4631	0.925855	2.02415	3.66445	0.20955404	3.16634112
Homo sapiens	Fused	five-degrees	18267	1.89735612	1.77939881	1.161775	1.8684	2.525125	3.56716	0.14626308	5.00175621
Hylobates lar	Fused	five-degrees	14186	1.27340807	1.13552726	0.63994	1.1822	1.8249	2.60764	0.35636072	4.11022603
Pongo abelii	Fused	five-degrees	25065	2.49890609	1.8090626	1.05685	1.9038	3.71065	5.981125	0.19160106	5.23682275
Gorilla gorilla	Non-fused	five-degrees	16069	2.30350088	1.85377965	1.02375	1.9645	3.27415	5.4456	0.17207357	5.97268124
Pan troglodytes	Non-fused	five-degrees	21714	1.06206996	0.77960554	0.33493	0.827685	1.6112	2.86634	0.13576369	6.16715284
Homo sapiens	Non-fused	five-degrees	14854	1.82079758	1.64781428	1.1241	1.711	2.4696	3.46846	0.46404682	3.83451694
Hylobates lar	Non-fused	five-degrees	14118	1.28548956	1.08535656	0.70702	1.11845	1.805	2.79166	0.82167145	3.04908478
Pongo abelii	Non-fused	five-degrees	25082	2.49322575	1.97851276	0.73315	2.05565	3.9171	5.9199	0.18946224	3.8987485
**Scaphoid**											
Gorilla gorilla	Fused	vertical	17700	2.0599	1.79535277	1.2214	1.86465	2.68105	4.1074	0.12735747	3.85981162

Pan troglodytes	Fused	vertical	19190	0.95097948	0.79587065	0.55972	0.818225	1.2567	1.9717	0.40575017	2.80879196
Homo sapiens	Fused	vertical	14283	2.3353	2.02127508	1.558625	2.3345	3.18405	3.8481	0.31982203	5.09729412
Hylobates lar	Fused	vertical	20329	0.84062567	0.61693069	0.40217	0.63085	1.1298	2.05622	0.83895328	2.25621906
Pongo abelii	Fused	vertical	21274	1.95390699	1.65045313	1.3309	1.80375	2.4232	3.77608	0.4618439	3.04474707
Gorilla gorilla	Non-fused	vertical	15913	3.88834987	3.40940301	2.357775	3.5358	5.092325	7.4499	0.5366978	3.70730555
Pan troglodytes	Non-fused	vertical	20517	2.50202603	1.98991665	1.215475	2.0548	3.516075	5.799465	0.55983057	3.26060629
Homo sapiens	Non-fused	vertical	11324	3.21128069	3.08961308	2.18135	3.18395	4.14465	5.56915	0.16397509	3.05335709
Hylobates lar	Non-fused	vertical	18138	1.53981773	1.31089261	0.80046	1.1397	1.6961	2.87246	0.17785139	2.59317472
Pongo abelii	Non-fused	vertical	18727	3.13032801	2.74593135	1.75085	2.8939	4.2145	6.22898	0.15679787	5.38865057
Gorilla gorilla	Fused	five-degrees	17697	2.05208279	1.78847376	1.216675	1.8571	2.670225	4.089865	0.10367777	3.83713967
Pan troglodytes	Fused	five-degrees	19190	0.94724402	0.79274189	0.55752	0.815015	1.2518	1.9639	0.40575493	2.80963003
Homo sapiens	Fused	five-degrees	14283	2.32686724	2.21331497	1.553225	2.3263	3.172325	3.834305	0.31983011	5.10478779
Hylobates lar	Fused	five-degrees	20329	0.83701784	0.61428533	0.4004425	0.62814	1.1249	2.04732	0.83894808	2.25541293
Pongo abelii	Fused	five-degrees	21274	1.94648955	1.74384802	1.3259	1.79695	2.414	3.76178	0.46184622	3.04510366
Gorilla gorilla	Non-fused	five-degrees	15913	3.87345893	3.39639002	2.3487	3.5223	5.072825	7.4214	0.53670094	3.70717069
Pan troglodytes	Non-fused	five-degrees	20517	2.49249694	1.9823561	1.21085	2.047	3.5027	5.777365	0.55982854	3.26096316
Homo sapiens	Non-fused	five-degrees	11324	3.19995407	3.07838052	2.17305	3.1734	4.1295	5.55266	0.16385877	3.08667111
Hylobates lar	Non-fused	five-degrees	18138	1.53464248	1.30660592	0.79737	1.1353	1.6895	2.86136	0.17785538	2.59298083
Pongo abelii	Non-fused	five-degrees	18727	3.1184445	2.73547647	1.74415	2.8829	4.1985	6.205295	0.1567978	5.38931808
**Centrale**											
Gorilla gorilla	Non-fused	vertical	5140	1.90526074	1.61213146	1.0902	1.7092	2.61395	3.86245	0.79500545	6.02113082
Pan troglodytes	Non-fused	vertical	7673	0.11701799	0.08379669	0.042244	0.088477	0.162595	0.326424	0.20454936	5.58531065
Homo sapiens	Non-fused	vertical	1623	0.30652851	0.26272318	0.1947825	0.27474	0.4058875	0.5802105	1.37451886	4.57394793
Hylobates lar	Non-fused	vertical	5823	0.68673613	0.48274929	0.2981225	0.49805	1.073425	1.635645	1.30189533	3.1694935
Pongo abelii	Non-fused	vertical	6833	1.53184785	1.41735316	1.0426	1.5141	2.067625	2.56908	0.45263187	6.82588106
Gorilla gorilla	Non-fused	five-degrees	5140	1.89796389	1.60596082	1.08605	1.7027	2.60395	3.84765	0.79500275	6.02375719
Pan troglodytes	Non-fused	five-degrees	7673	0.11657234	0.08347813	0.04208325	0.08814	0.161975	0.325184	0.20454984	5.58454586

Homo sapiens	Non-fused	five-degrees	1623	0.30551631	0.2611057	0.1957575	0.27365	0.4047125	0.5761325	1.35714752	4.80429786
Hylobates lar	Non-fused	five-degrees	5823	0.68408326	0.48087363	0.29697	0.49613	1.06925	1.629345	1.30189626	3.17263656
Pongo abelii	Non-fused	five-degrees	6833	1.52603223	1.41193655	1.038675	1.5084	2.059725	2.55938	0.45262554	6.8319962

Key, MWAM; mesh-weighted average mean, MWM; mesh-weighted median, M(25); 25% percentile, M(50); 50% percentile, M(75); 75% percentile, M(95); 95% percentile, PEofAM; percentage error of the arithmetic mean, PEofM; percentage error of the medi
